# Molecular and oral manifestations of langerhans cell histiocytosis preceding acute myeloid leukemia

**DOI:** 10.1186/s12903-022-02410-z

**Published:** 2022-09-05

**Authors:** Qi Zhang, Xiaoting Wu, Xiaobo Wang, Evenki Pan, Li Ying

**Affiliations:** 1 Department of Hematology, The Second Hospital of Dalian Medical University, Dalian, China; 2 The Third Clinical Medical College, Zhejiang Chinese Medical University, Hangzhou, China; 3 Nanjing Geneseeq Technology Inc., Nanjing, China; 4 Department of Gastroenterology, The Second Hospital of Dalian Medical University, Dalian, China

**Keywords:** Histiocytosis, Langerhans-cell, Leukemia, Mutation, Case reports

## Abstract

**Background:**

Langerhans cell histiocytosis (LCH) is a heterogeneous neoplastic disorder that is rarely seen in patients aged 60 years and older. It is reported that elderly patients with LCH have a higher chance of having malignancies. In the oral cavity, patients with LCH can present with mucosal ulcers and extensive osteolysis, making it difficult for clinicians to make a proper diagnosis.

**Case presentation:**

We reported an 82-year-old Chinese woman with oral symptoms as the first presentation of LCH, and eventually developed acute myeloid leukemia (AML). She suffered diffuse ulcers involving the entire gingival mucosa and the left half hard palate, and had lost several teeth. Genomic DNA sequencing of the cells from LCH revealed multiple mutations in *TET2, BRAF, SRSF2, NRAS, MAP2K4* and so on. The patient declined the *BRAF*^*V600E*^ inhibitor (Vemurafenib). Although a dramatic improvement of the oral ulcers was achieved after symptomatic treatment, the patient developed acute myeloid leukemia (AML) and died.

**Conclusions:**

This report presented the diagnostic difficulties of LCH with oral manifestations and highlighted the importance of radiological assessments and laboratory tests. Moreover, many of the mutations detected in our LCH patient are frequently seen in AML, suggesting that AML and LCH cells in this patient share the same origin.

## Background

Langerhans cell histiocytosis (LCH) is an inflammatory myeloid neoplasm, it is rarely diagnosed in adults (1–2 per million adults per year), even fewer in people over 60 years of age [1]. The clinical presentations are highly heterogeneous, ranging from a solitary lesion to multisystem involvement that is associated with organ dysfunction, which can affect the bones, skin, lungs and hematopoietic system, etc. Patients with oral LCH can present as mucosal ulcers, gingival hyperplasia, jaw necrosis, and dental luxation or loss [2]. These clinical manifestations are difficult to distinguish from those of periodontitis or gingivitis. Definitive diagnosis can be made after histopathological examination.

The constitutive activation of the mitogen-activated protein kinase (MAPK) pathway is the hallmark of LCH, making it a target of therapeutic agents [3]. The current understanding is that LCH are likely arises from the abnormal differentiation or recruitment of hematopoietic precursors, supporting the hypothesis that LCH is the result of misguided myeloid differentiation [4]. It is reported that LCH can occur before or after, or concurrent with myeloid neoplasms such as acute myeloid leukemia (AML), suggesting the two entities are clonally related [5]. The unclear etiopathogenesis and wide clinical spectrum pose great challenges to diagnosis, treatment and prognosis. We herein reported a unique case of LCH in which the patient first presented with oral ulcers and later developed AML. Genomic mutations in the LCH cells were studied and discussed.

## Case presentation

**Fig. 1:**
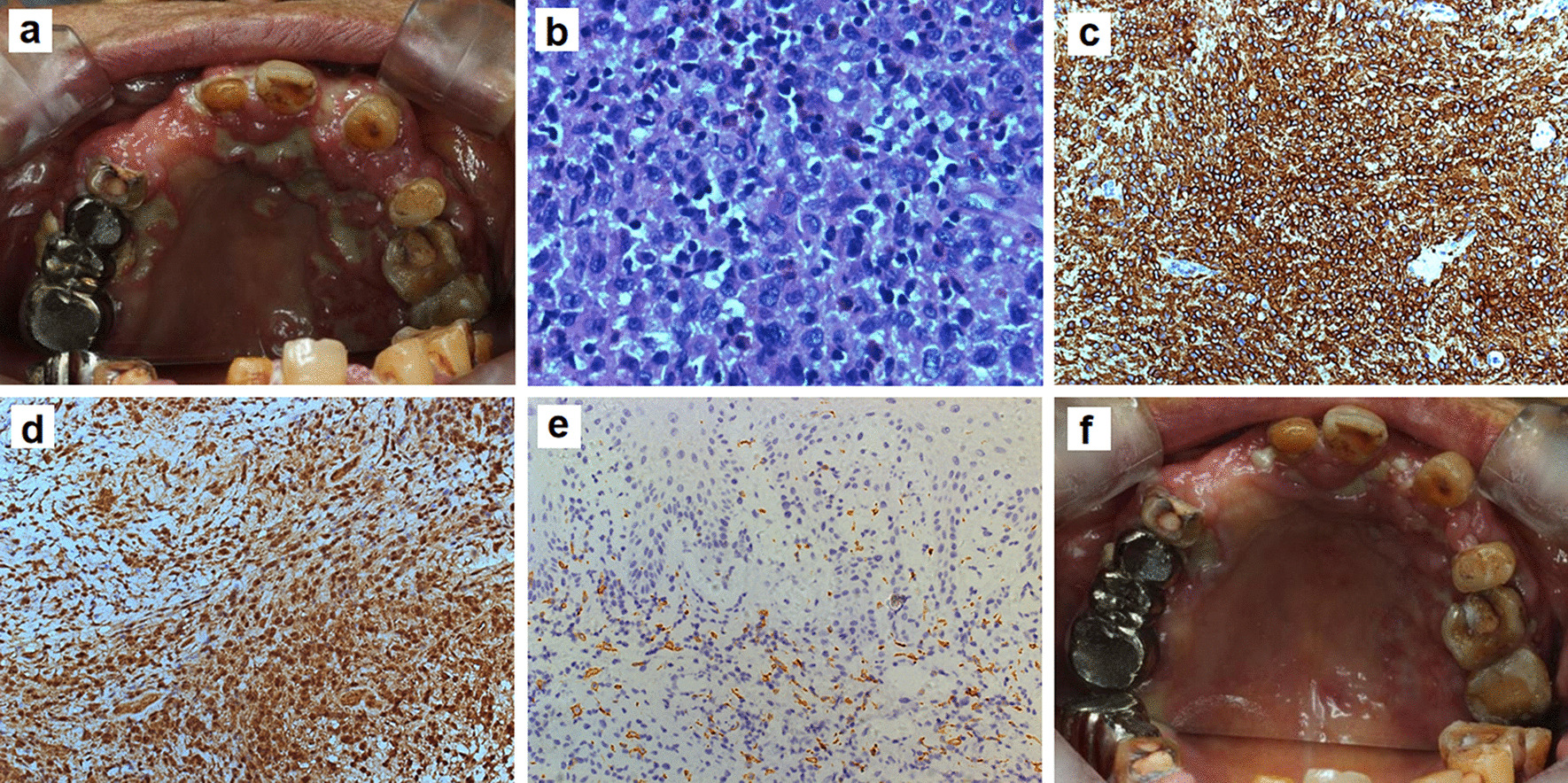
Clinical presentation and histopathologic findings of LCH. **a** Intraoral examination shows multiple ulcers and suppurations on the whole gingival mucosa and left hard-palate, with teeth loss; **b** Skin biopsy shows the intense proliferation of histiocytes, significant eosinophils and lymphocytes infiltrating (HE, × 600); Positive immunohistochemistry for **c** CD-1a, **d** S-100 and **e** CD163 in oral tissue (IHC, × 200); **f** Oral condition after one month of treatment *HE* Hematoxylin and eosin stain; *IHC* Immunohistochemistry stain

**Fig. 2:**
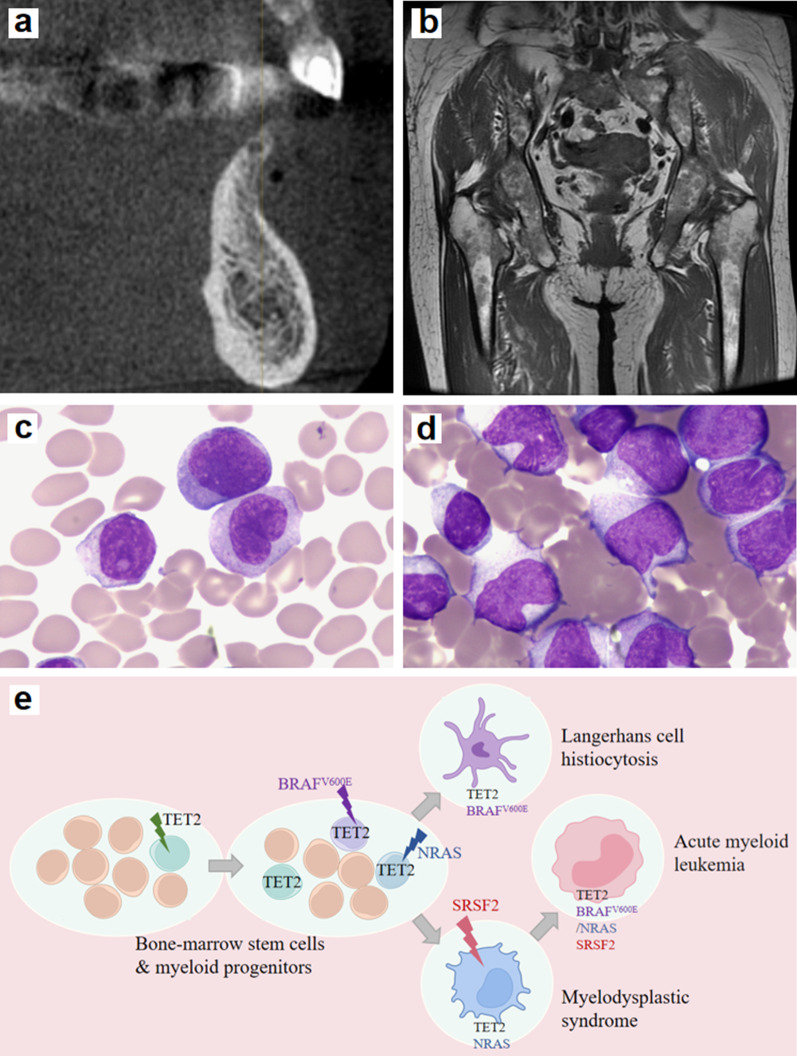
Imaging findings and cell morphology findings. **a** X-ray shows bone destruction. **b** MRI shows inhomogeneous signal intensity at pelvic bones, lumbosacral vertebrae and bilateral femurs. Cell morphology test shows myeloblasts and promonocytes in **c** the peripheral blood and **d** bone marrow increased. **e** The hypothesized order for the appearance of mutations

An 82 year-old woman presented to our clinic complaining of severe oral ulcerations that caused difficulty eating. Intraoral examination showed diffuse ulcers with suppurative discharge involving the entire gingival mucosa and the left half hard palate, multiple teeth were lost (Fig. 1a). A biopsy was performed, pathology showed an intense proliferation of histiocytes and significant infiltration of eosinophils and lymphocytes (Fig. 1b). CD-1a, S-100 and CD163 were highlighted on immunohistochemistry staining (Fig. 1c–e). Bone destruction was found by oral and maxillofacial radiology (Fig. 2a), and hip magnetic resonance imaging (MRI) revealed inhomogeneous signal intensity at pelvic bones, lumbosacral vertebrae and bilateral femurs (Fig. 2b). A blood count showed WBC leucocytes 7.85 × 10^9^/L, RBC 3.3 × 10^12^/L, hemoglobin 112 g/L and platelets 175 × 10^9^/L. A bone marrow biopsy was performed, flow cytometry identified slight developmental anomaly in primitive myeloid cell with increased monocytes (21.01%), there was granulocyte dysplasia. The diagnosis of LCH with myelodysplastic syndrome (MDS) was made. Genomic sequencing was performed in the LCH cells, which revealed mutations in *TET2, BRAF, SRSF2, NRAS, MAP2K4, MPL, DDX41,*and *MSH3* (Table 1).

**Table 1:** Gene mutations detected by next-generation sequencing at the tissue wax of the patient

Gene	Amino acid change	Coding	Exon	Variant effect	Allele frequency	Transcript	Locus	Protein change	MIM number	Clinical significance	Phenotype
TET2	p.Q1540*	c.4618C > T	11	Truncated mutation	40.5	NM_001127208.3	–	–	612,839	Likely oncogenic	Chronic Myelomonocytic Leukemia, Myelodysplastic Syndromes, Angioimmunoblastic T-Cell Lymphoma
TET2	p.R131Kfs*5	c.391dup	3	Frameshift mutation	28.6	NM_001127208.3	–	–	612,839	Likely oncogenic	
BRAF	p.V600E	c.1799 T > A	15	Missense mutation	17.0	NM_004333.6	chr7:140,753,335	V600E	164,757	Oncogenic	Anaplastic Thyroid Cancer, Colorectal Cancer, Melanoma, Non-Small Cell Lung Cancer, Biliary Tract Cancer, Glioma, Astrocytoma, Leukemia
SRSF2	p.P95H	c.284C > A	1	Missense mutation	3.2	NM_001195427.2	–	–	600,813	Oncogenic	Acute Myeloid Leukemia
NRAS	p.G12D	c.35G > A	2	Missense mutation	0.8	NM_002524.5	chr1:114,716,126	G12D	164,790	Oncogenic	Colorectal Cancer, Erdheim-Chester Disease, Langerhans Cell Histiocytosis, Rosai-Dorfman Disease, Histiocytosis, Melanoma, Thyroid Cancer
MAP2K4	p.R145W	c.433C > T	5	Missense mutation	3.2	NM_003010.4	–	–	601,335	Unknown	–
MPL	p.W632C	c.1896G > C	12	Missense mutation	5.0	NM_005373.3	–	–	159,530	Unknown	Myelofibrosis with myeloid metaplasia, Thrombocythemia 2, Amegakaryocytic Thrombocytopenia
DDX41	p.R311Q	c.932G > A	9	Missense mutation	1.1	NM_016222.4	–	–	608,170	Unknown	Myeloproliferative/Lymphoproliferative Neoplasms
MSH3	p.H781Y	c.2341C > T	17	Missense mutation	15.6	NM_002439.5	chr5:80,778,742	H781Y	600,887	Unknown	Endometrial Carcinoma, Familial Adenomatous Polyposis 4

The patient declined chemotherapy or *BRAF*^*V600E*^ inhibitor (Vemurafenib), she was treated with dexamethasone mouth rinse, thalidomide (100 mg/day) and prednisone (30 mg/day). One month later, a dramatic improvement of the oral ulcers was achieved, and the patient could eat normally (Fig. 1f). Thalidomide and prednisone were tapered. However, three months later, she developed bone ache, fatigue, and shortness of breath. The blood routine examination was performed: WBC 74.04 × 10^9^/L, RBC 2.98 × 10^12^/L, hemoglobin 92 g/L, platelets 10 × 10^9^/L. There was significant increase of myeloblasts and promonocytes in the peripheral blood and the bone marrow (Fig. 2c–d), consistent with the development of AML. The patient decided to pursue comfort care and expired a month later.

## Discussion and conclusion

The clinical features of oral LCH are mainly swelling, pain, mucosal ulceration, and gingival hyperplasia, and tooth loss [2]. Oral soft tissue lesions usually occur in conjunction with jaw lesions, which affect the alveolar bone and lend to dental luxation or loss. In addition, LCH can mimic the clinical symptoms of other conditions, including viral infection, malignancy, periodontal and granulomatous diseases [4]. Differentiating LCH from these is challenging and a dentist may miss the underlying diagnosis. In the oral cavity, patients with LCH occasionally present with painful mucosal ulcers and dental loss, which may be misdiagnosed as periodontitis. Positive immunohistochemical stains of CD-1a and S-100 are the characteristic biomarkers of LCH. Definitive diagnosis can be made after careful radiological and histopathological examination. Although patients with oral LCH have limited oral lesions, they are at risk of systemic involvement [6]. Therefore, once a patient has been diagnosed with LCH, a thorough physical examination should be performed to rule out other diseases, especially malignancies. Therefore, it is necessary to perform radiological assessment and a tissue biopsy to ensure the diagnostic accuracy, LCH has been mainly diagnosed in children, rarely in adults, even fewer in people over 60 years of age.

It has been observed that adult patients with LCH have a higher incidence of malignancies, the exposure to chemotherapeutic agents such as etoposide has been thought to have contributed to the carcinogenesis [7]. However, our patient did not receive such agents for LCH treatment, but still developed AML. Consistent with that, about 32% of adult LCH patients who had not received etoposide were diagnosed with secondary malignancies, including solid tumors, lymphomas, and hematologic malignancies, arguing other causes [1]. which indicated the occurrence of malignancy in patients with LCH has irrelevance to its treatment, but the mechanism of the association between adult LCH and malignancy has not been elucidated. In this report, the gene mutations in the clonal evolution of the patient deserved consideration.

On the one hand, some mutations related with MAP kinase (such as *BRAF*, *MAP2K4*, *NRAS*) were found in our patient (Table 1). BRAF, an intracellular kinase, is frequently mutated in melanoma, thyroid and lung cancers among others. The variant residue at sequence position 600 in this protein is a glutamic acid which has a negatively charged side chain, making it hydrophilic. *BRAF*^*V600E*^ mutation is a constitutive activator of the MAPK pathway that promotes cell proliferation, apoptosis and invasion by activating the downstream MEK-ERK transduction pathway, which is involved in more than 50% of LCH lesions [8, 9]. Besides, *MPL* and *MAP2K4* mutations can also activate MAPK and/or PI3K/AKT signaling pathways and promote malignant transformation [10, 11]. MPL, a transmembrane protein receptor, is frequently mutated in myeloproliferative neoplasms including essential thrombocytosis and AML [10]. The variant residue at sequence position 632 is a cysteine which has a side chain capable of forming a disulphide bond with another cysteine, and hence provide a strong structural support for the protein. MAP2K4 is a tumor suppressor and intracellular kinase that has been reported to be associated with histiocytic neoplasms [11]. The variant residue at sequence position 145 in this protein is a tryptophan which has an aromatic side chain. Of note, Zhang J et al. [12] found another *MAPK* (*MAP3K15*) gene mutation in a girl who developed acute lymphoblastic leukemia after LCH, and they suggested that *MAPK* gene may be a potential biomarker for the conversion of LCH to haematological malignancies, which was consistent with our findings. In addition, *N/KRAS* mutation detected in histiocytic neoplasms were also seen in myeloid leukaemias, suggesting the common hematopoietic stem/progenitor cells they shared [13]. This mutation is closely associated with myeloblast development and could be the predictor of evolution to clonally relevant haematological malignancies [4, 13]. Hence, genomic sequencing analysis of patients with histiocytosis is helpful in clarifying the staging and providing positive early intervention [12, 13]. The patient also had a mutation in *DDX41* gene, which is associated with myeloid neoplasm including MDS, AML and CMML, but rarely with LCH [14].

On the other hand, some mutations related to epigenetic regulation (such as *TET2*, *SRSF2*) were also found (Table 1), most of them have been described to participate in the pathogenesis of AML. The residue at sequence position 1540 and 131 in TET2 protein were altered and these variants may be closely related to the development of hematologic malignancies. SRSF2 is an RNA splicing factor that is frequently mutated in hematological malignancies, and its variant residue is a histidine with a positively charged side chain, making it hydrophilic. It was reported that *SRSF2* and *TET2* mutations were detected in LCH cells of a patient with concurrent mixed histiocytosis and acute myelomonocytic leukemia (AMML), it showed that *TET2* mutation could occur before *BRAF* and *SRSF2* mutations in vitro [15]. MDS patients with *SRSF2* mutation had inferior 5 year overall survival than those without that, leading to a faster evolution into AML [16]. Yoshimi A reported that co-mutations in *SRSF2* and *IDH2* can drive myeloid malignancy development through promoting lethal myelodysplasia in vivo [17] and revealed a pathogenic cross-talk between the epigenome and RNA splicing in AML. These findings also suggest that mutations in AML are not isolated.

Two-hit hypothesis supports that the occurrence of AML is a multi-step process consisting of a wide range of genetic and phenotypic changes [18]. Generally, mutations as the first-hit do not directly give rise to AML, the coordination with order with other mutations made the transformation into a full-blown AML eventually. Lindsley et al. [19] have analyzed eight specific gene mutations related to secondary AML (s-AML) and found the most frequent were *ASXL1* and *SRSF2* mutations which occurred at 32% and 20% frequency, respectively. As for the order in which mutations appear, many researchers supported those gene mutations involving DNA methylation, chromatin modification, and RNA splicing appear before others, followed by transcription related, while mutations associated with the tyrosine kinases and RAS signaling pathways are often the last event in clonal evolution [17]. What’s more, there are some reports on the mutual transformation of LCH and AML, in which researchers have proposed that this concomitant was owing to the common tumour hematopoietic stem cell they shared, but the specific genetic machinery was inconclusive [2]. In our case, the presence of *TET2, BRAF, SRSF2, NRAS, MAP2K4, MPL, DDX41, MSH3* mutations might provide an explanation for the clonal evolution of LCH and AML: *TET2* mutation as the first-hit appeared first, the subsequent *BRAF* and *NRAS* mutations initiated the occurrence of LCH and MDS, finally the *SRSF2* mutation and the former together drove an AMLtransformation (Fig. 2e).

In conclusion, this report descripted an elderly female with oral symptoms as the first presentation of LCH, and eventually developed AML. We highlighted the necessity of radiological and histopathological examinations in the diagnosis of oral LCH, and the importance of genetic testing for early intervention, early treatment and management of LCH patient. Moreover, the mutation profiles suggests that LCH and AML might have a common clonal origin, and provided hypothesis of the development of LCH and AML.

## Data Availability

Not applicable.

## Abbreviations

LCH: Langerhans cell histiocytosis
MDS: Myelodysplastic syndrome
AML: Acute myeloid leukemia
AMML: Acute myelomonocytic leukemia
